# A diagnostic accuracy study validating cardiovascular ICD-9-CM codes in healthcare administrative databases. The Umbria Data-Value Project

**DOI:** 10.1371/journal.pone.0218919

**Published:** 2019-07-08

**Authors:** Francesco Cozzolino, Alessandro Montedori, Iosief Abraha, Paolo Eusebi, Chiara Grisci, Anna Julia Heymann, Guido Lombardo, Anna Mengoni, Massimiliano Orso, Giuseppe Ambrosio

**Affiliations:** 1 Health Planning Service, Regional Health Authority of Umbria, Perugia, Italy; 2 Division of Cardiology, Santa Maria della Misericordia Hospital, University of Perugia School of Medicine, Perugia, Italy; 3 Centro Regionale Sangue, Servizio Immunotrasfusionale, Azienda Ospedaliera di Perugia, Perugia, Italy; 4 Department of Experimental Medicine, Section of Public Health, University of Perugia, Perugia, Italy; 5 Istituto Zooprofilattico Sperimentale dell'Umbria e delle Marche “Togo Rosati”, Perugia, Italy; 6 Department of Surgical and Biomedical Sciences, University of Perugia, Perugia, Italy; Universita degli Studi di Firenze, ITALY

## Abstract

**Background:**

Administrative healthcare databases are useful and inexpensive tools that can provide a comprehensive assessment of the burden of diseases in terms of major outcomes, such as mortality, hospital readmissions, and use of healthcare resources. However, a crucial issue is the reliability of information gathered. The aim of this study was to validate ICD-9 codes for several major cardiovascular conditions, i.e., acute myocardial infarction (AMI), atrial fibrillation/flutter (AF), and heart failure (HF), in order to use them for epidemiological, outcome, and health services research.

**Methods:**

Data from the centralised administrative database of the Umbria Region (890,000 residents, located in Central Italy) were considered. Patients with a first hospital discharge for AMI, AF/flutter, and HF, between 2012 and 2014, were identified using ICD-9-CM codes in primary position. A sample of cases and non-cases was randomly selected, and the corresponding medical charts reviewed by specifically trained investigators. For each disease, case ascertainment was based on all clinical, laboratory, and instrumental examinations available in medical charts. Sensitivity, specificity, and predictive values with 95% confidence intervals (CIs), were calculated.

**Results:**

We reviewed 458 medical charts, 128 for AMI, 127 for AF/flutter, 127 for HF, and 76 of non-cases for each condition. Diagnostic accuracy measures of the original discharge diagnosis were as follows. AMI: sensitivity 98% (95% CI, 94–100%), specificity 91% (95% CI, 83–97%), positive predictive value (PPV) 95% (95% CI, 89–98%), negative predictive value (NPV) 97% (95% CI, 91–100%). AF/flutter: sensitivity 95% (95% CI, 90–98%), specificity 95% (95% CI, 87–99%), PPV 97% (95% CI, 92–99%), NPV 92% (95% CI, 84–97%). HF: sensitivity 96% (95% CI, 91–99%), specificity 90% (95% CI, 81–96%), PPV 94% (95% CI, 88–97%), NPV 93% (95% CI, 85–98%).

**Conclusion:**

The case ascertainment for AMI, AF and flutter, and HF, showed a high level of accuracy (≥ 90%). The healthcare administrative database of the Umbria Region can be confidently used for epidemiological, outcome, and health services research.

## Introduction

Administrative databases are considerable data repositories that are increasingly being used within healthcare systems[1, 2]. These databases are organized and maintained at different administrative levels including hospitals, local health units, and at regional level. The continuous collection of demographic data together with diagnosis, therapeutic interventions as well as prescription information makes these databases attractive for comprehensive assessment of the burden of diseases in terms of major outcomes, such as mortality, hospital readmissions, and use of healthcare resources[1, 2].

In addition to maintaining a rigorous anonymity of patient’s demographic, the most relevant data that makes these healthcare databases interesting for research purposes is the diagnosis provided to the patient at hospital discharge. This diagnosis is coded according to the International Classification of Diseases (ICD) which is a standardized diagnostic tool planned to map health conditions. When individual patient data are linked with other data (prescription and laboratory data) it is possible to explore a wide range of clinical issues, research questions as well as quality performance evaluations. To reach this target, administrative databases need to be validated, which means the diagnoses that correspond to the ICD-9 code need to be ascertained according to a defined disease criteria by consulting a reference standard which is usually the medical chart[1, 3, 4].

Acute myocardial infarction (AMI), heart failure (HF), and atrial fibrillation (AF) are the most common cardiovascular diseases[5–7] in developed countries and they are the leading cause of morbidity and mortality, thereby representing a major social and economic problem[5, 8]. Administrative databases are significant tools that can provide the best assessment of the incidence, prevalence and general prognosis of cardiovascular diseases[3, 9–11]. In addition, they can contribute to identifying the risk factors in the development of cardiovascular diseases as well as the outcomes, including mortality, that they can determine.

According to our published protocol[3], the objective of the present study was to evaluate the accuracy of the ICD-9-CM codes related to AMI, AF and flutter, and HF in the administrative database of the Regional Health Authority of Umbria.

## Materials and methods

### Setting and data source

#### Administrative database and source population

Details regarding the regional healthcare administrative database of Umbria have been reported elsewhere[3]. Briefly, this database constantly collects demographic and hospital data as well as prescription data regarding all residents. The Umbria administrative healthcare database has been used for research purposes[12–15] and it has been validated for several oncological diagnoses[16–21].

Any permanent resident in the Umbria Region aged 18 years or older that has been discharged from a hospital with the diagnosis of AMI, AF or atrial flutter, and HF was eligible for inclusion.

Residents that have been hospitalised outside the regional territory of Umbria were excluded from analysis[3].

#### Case selection and sampling method

From the entire discharge abstract database of Umbria we identified three cohorts of “cases”, that is patients having the ICD-9 codes located in primary position of acute myocardial infarction (ICD-9 codes 410.x), atrial fibrillation (code 427.31) and flutter (code 427.32), and heart failure (codes 428.x), between 2012 and 2014. According to Italian legislation, the primary diagnosis constitutes the main cause of the need for treatment and/or diagnostic tests, and is mainly responsible for the use of resources. We excluded estimated prevalent cases, i.e. patients discharged from hospitals with the same diagnosis in the five years before. Repeated hospital admissions were also excluded. From each cohort, we extracted a random sample of 130 cases (see Statistical Methods for details). At the same time, we identified a cohort of “non-cases”, that is patients who had been discharged in the same period of time, also with a diagnosis of cardiovascular disease (ICD-9 codes 390–459), but other than AMI, AF and flutter, and HF. From this cohort of non-cases, we extracted a random sample of 80 patients. This sample of non-cases was used as control for each of the three conditions.

### Chart abstraction and case ascertainment

Medical charts of the samples of cases and non-cases were selected by simple randomization method using SAS 9.4 procedures and were obtained from hospitals for case ascertainment. The following data were abstracted from the medical charts: unique patient identification code, age and sex, hospital admission and discharge dates, diagnostic procedures as well as interventions that contributed to the diagnosis of the disease. In addition, clinical, laboratory and instrumental procedures data, including the date of performance, were abstracted.

Two physicians acting as chart reviewers received specific training on data abstraction. An initial chart review was then performed, with the same medical charts (n = 20) being independently examined by each reviewer. The inter-reviewer agreement was very high (κ >0.9). To further ensure consistency among the reviewers, findings of this initial assessment were discussed in review; data extraction was then completed independently by each reviewer using a standardised data collection form. Reviewers were not blinded to the diagnosis due to limitation of resources. However, the validation process was not biased as the reviewers independently abstracted the data.

Uncertainties were discussed and resolved through third party involvement (GA).

Case ascertainment of disease within medical charts was based on symptoms, laboratory, and diagnostic tests, as described below.

### Validation criteria

Criteria used for validation of the considered ICD-9-CM codes were those derived from international guidelines or systematic reviews published on these topics, as previously defined [3]. To validate ICD-9 codes 410.x for AMI, we considered the criteria defined by the European Society of Cardiology [22].

For validation of the ICD-9 code 427.31 for AF and 427.32 for atrial flutter, we required at least one ECG tracing documenting, the presence of AF or atrial flutter, according to the ESC Guidelines [23].

To validate the ICD-9 codes 428.x relating to HF, we considered the European Society of Cardiology Heart Failure Guidelines [24]. These guidelines consider the algorithm for the diagnosis of HF in the non-acute setting and in the acute setting. As the ICD-9 codes do not distinguish between acute and non-acute settings, we combined the clinical presentation of both settings. Accordingly, diagnosis of HF was adjudicated when, in addition to the presence of symptoms (such as dyspnoea, orthopnoea), or presence of signs at physical examination (rales, bilateral ankle oedema, increased jugular venous pressure, displaced apical beat), at least one of the following conditions were found in the medical chart: 1. any abnormalities in resting ECG; 2. brain natriuretic peptide (BNP) concentration ≥35 pg/mL, and/or N-terminal pro-brain natriuretic peptide (NT-proBNP) ≥125 pg/mL); 3. echocardiography abnormalities (ventricular and atrial volumes and function) attributable to heart failure.

### Statistical analysis

As reported elsewhere[3], for each index condition (ICD-9 code) we anticipated a sample of 121 charts of cases to obtain an expected sensitivity of 80%, with a half-width of the 95% CI equals to 8% [25]. For specificity, we calculated that a sample of 73 charts of non-cases (i.e., cardiovascular patients without the target diseases of our interest) was necessary to obtain an expected specificity of 90%, with a half-width of the 95% CI equals to 8%, according to binomial exact calculation [25]. Expected accuracy figures were based on published literature [10, 11, 26–29]. We then chose to review a higher number of medical charts compared to the anticipated sample size to allow for potential missing medical charts from hospital archives.

Sensitivity and specificity, with their corresponding 95% CI, were calculated for each ICD-9-CM code by producing 2×2 tables. Positive and negative predicting values were also calculated, along with their 95% CI.

### Reporting

To ensure quality and thoroughness of reporting, this paper followed the criteria of Standards for Reporting Diagnostic Accuracy (STARD) 2015 [30] (S1 Table).

### Ethics statement

Ethics approval has been obtained from the Regional Ethics Committee of Umbria (CEAS), registry No 2695/15 of 16/12/2015.

## Results

From each cohort, we extracted a random sample of 130 cases, while from the cohort of non-cases we extracted a random sample of 80 patients. Of these, we were able to retrieve and analyse 128 medical charts for AMI, 127 for AF and flutter, and 127 for HF. For non-cases, we retrieved and analysed 76 medical charts. **Tables 1–3** show the characteristics of the patients for each disease. A minimal anonymized dataset is available as supporting information file (S1 Dataset).

**Table 1:** Characteristics of patients with acute myocardial infarction who were identified in the Regional Administrative Database of Umbria.

**Acute myocardial infarction**	
**Incident cases** (N medical charts reviewed)	128
**ICD-9 code, N (%) **	
410.0 Anterolateral wall	3 (2)
410.1 Other anterior wall	31 (24)
410.2 Inferolateral wall	8 (6)
410.3 Inferoposterior wall	1 (1)
410.4 Other inferior wall	22 (17)
410.5 Other lateral wall	1 (1)
410.6 True posterior wall infarction	-
410.7 Subendocardial infarction	53 (41)
410.8 Other specified sites	1 (1)
410.9 Unspecified site	8 (6)
**Sex, N (%) **	
Male	85 (66)
Female	43 (34)
**Age, N (%) **	
*< 60*	29 (23)
*60–79*	61 (48)
*≥ 80*	38 (30)
**Instrumental examinations, N (%) **	
Electrocardiography (ECG)	125 (98)
Echocardiography	110 (86)
Coronary arteriography	91 (71)
**Laboratory analyses, N (%)**	
Troponin	124 (97)
CPK-MB	60 (47)
Creatine phosphokinase (CPK)	28 (22)
Lactate dehydrogenase (LDH)	31 (24)
**Surgical procedures, N (%) **	
Percutaneous transluminal coronary angioplasty (PTCA) or coronary atherectomy	79/128 (62)
PTCA in STEMI	54/79 (68)
PTCA in NSTEMI	25/79 (32)

**Table 2:** Characteristics of patients with atrial fibrillation and flutter who were identified in the Regional Administrative Database of Umbria.

**Atrial fibrillation and flutter **	
**Incident cases** (N medical charts reviewed)	127
**ICD-9 code, N (%)**	
427.31 Atrial fibrillation	107 (84)
427.32 Atrial flutter	20 (16)
**Sex**	
Male	67 (53)
Female	60 (47)
**Age, N (%) **	
< 60	14 (11)
60–79	66 (52)
≥ 80	47 (37)
**Instrumental examinations, N (%) **	
Electrocardiography (ECG)	126 (99)
Echocardiography	92 (72)
**Procedures, N (%)**	
Electrical cardioversion	37 (29)

**Table 3:** Characteristics of patients with heart failure who were identified in the Regional Administrative Database of Umbria.

**Heart failure**	
**Incident cases** (N medical charts reviewed)	127
**ICD-9 code, N (%)**	
428.0 Congestive heart failure, unspecified	81 (64)
428.1 Left heart failure	30 (24)
428.2 Systolic heart failure	
428.20 Unspecified	-
428.21 Acute	-
428.22 Chronic	-
428.23 Acute on chronic	3 (2)
428.3 Diastolic heart failure	
428.30 Unspecified	2 (2)
428.31 Acute	-
428.32 Chronic	-
428.33 Acute on chronic	1 (1)
428.4 Combined systolic and diastolic heart failure	
428.40 Unspecified	-
428.41 Acute	3 (2)
428.42 Chronic	-
428.43 Acute on chronic	5 (4)
428.9 Heart failure, unspecified	2 (2)
**Sex, N (%)**	
Male	60 (47)
Female	67 (53)
**Age, N (%)**	
< 60	4 (3)
60–79	45 (35)
≥ 80	78 (61)
**Instrumental examinations, N (%)**	
Electrocardiography (ECG)	123 (97)
Echocardiography	81 (64)
Chest radiography	61 (48)
Coronary arteriography	10 (8)
**Laboratory analysis, N (%)**	
Brain natriuretic peptide (BNP)	59 (46)

Diagnostic accuracy measures are reported in **Table 4**.

**Table 4:** Cross tabulation of the index test (ICD-9-CM code) for the results of the reference standard (medical chart).

	True Positive	False Positive	True Negative	False Negative
Myocardial infarction	121	7	74	2
Atrial fibrillation and flutter	123	4	70	6
Heart failure	119	8	71	5

### Acute myocardial infarction

We identified a cohort of 4,682 patients with AMI, from which we extracted a sample of 130 cases, 128 clinical charts were analysed (two clinical charts were not available). **Table 1** shows the basic characteristics of the patients with AMI. The most common ICD-9 subgroup was code 410.7 (subendocardial infarction) (41%), followed by code 410.1 (other anterior wall) (24%), and by code 410.4 (other inferior wall) (17%). Two thirds of patients were males. Most patients (48%) were in the age class 60–79 years. Instrumental examinations performed for the diagnosis included electrocardiography, echocardiography, and coronary angiography. Troponin levels were measured in almost all patients. Sixty-two percent of patients underwent percutaneous transluminal coronary angioplasty (PTCA).

**Table 2** shows the diagnostic accuracy measures for AMI: sensitivity 98% (95% CI: 94% - 100%), specificity 91% (95% CI: 83% - 97%), positive predictive value (PPV) 95% (95% CI: 89% - 98%), and negative predictive value (NPV) 97% (95% CI: 91% - 100%).

Misclassification of cases and non-cases is described in **Table 5**. False positives were patients with troponin levels within the normal range, or not reported in the medical chart. The false negatives were non-cases actually found to have increased troponin concentration and presence of symptoms or instrumental confirmation of myocardial infarction.

**Table 5:** Reasons for incorrect identification of cases and controls.

	Myocardial infarction	Atrial fibrillation and flutter	Heart failure
**FALSE POSITIVES**	- Troponin levels not reported with the presence of signs or symptoms, or instrumental confirmation of myocardial infarction (n. 3); - Troponin levels not reported without the presence of signs or symptoms, or instrumental confirmation of myocardial infarction (n. 1); - Troponin levels within the normal range with the presence of signs or symptoms, or instrumental confirmation of myocardial infarction (n. 3).	- Atrial fibrillation in the medical history without the presence in the medical chart of an ECG confirmation (n. 4).	- Signs and symptoms of heart failure without instrumental or laboratory confirmation in the medical chart (n. 7); - Patient with instrumental confirmation of heart failure without signs or symptoms, admitted for implantation of cardiac resynchronization defibrillator (n. 1).
**FALSE NEGATIVES**	- Patients with altered troponin levels and the presence of symptoms with instrumental confirmation of myocardial infarction (n. 2).	- Patients with an ECG confirmation of atrial fibrillation or flutter (n. 6).	- Patients with signs and symptoms with instrumental confirmation of heart failure (n. 5).

### Atrial fibrillation/flutter

We identified a cohort of 2,792 patients with AF/flutter, from which we extracted a sample of 130 cases, 127 clinical charts were analysed (three clinical charts were not available).

**Table 3** shows the basic characteristics of the patients with AF/flutter. In this sample, 84% percent of patients had atrial fibrillation, while 16% had atrial flutter according to ICD codes in primary position. Fifty-three percent of patients were males. Most patients (52%) were in the age class 60–79 years. During hospital stay, almost all patients underwent electrocardiography, and most of them (72%) echocardiography. Twenty-nine percent of the patients underwent electrical cardioversion (**Table 2**).

The diagnostic accuracy measures for AF/flutter were: sensitivity 95% (95% CI: 90% - 98%), specificity 95% (95% CI: 87% - 99%), PPV 97% (95% CI: 92% - 99%), and NPV 92% (95% CI: 84% - 97%) **(Table 2)**.

All patients without an ECG in the medical chart, or with ECG negative for AF/flutter, were considered false positives. All false positives had a clinical history of AF or flutter, while, between the non-cases, false negatives were patients having other cardiovascular diseases even with an ECG confirmation of AF or flutter in the medical chart (**Table 5**).

### Heart failure

We identified a cohort of 6,196 patients with AMI, from which we extracted a sample of 130 cases, 127 clinical charts were analysed (three clinical charts were not available).

**Table 4** shows the basic characteristics of patients with HF. The most common ICD-9 subgroup was code 428.0 (congestive heart failure, unspecified) (64%), followed by code 428.1 (left heart failure) (24%). Fifty-three percent of patients were females. Most patients (61%) were 80 years or older. The most frequent diagnostic instrumental examinations were electrocardiography, echocardiography, and chest radiography. Brain natriuretic peptide (BNP) levels were measured in 46% of patients (**Table 3**).

The diagnostic accuracy measures for HF were: sensitivity 96% (95% CI: 91% - 99%), specificity 90% (95% CI: 81% - 96%), PPV 94% (95% CI: 88% - 97%), and NPV 93% (95% CI: 85% - 98%) (**Table 2**).

Patients with signs or symptoms but without instrumental or laboratory confirmation of HF in their medical chart were considered false positives. Most false positives (5/8) were patients ≥ 85 having polypathologies that during the hospital admission underwent only treatment procedures, no instrumental examinations were performed. Among the non-cases, false negatives were patients that, in addition to other cardiovascular diseases, also had signs, symptoms and instrumental examinations confirming heart failure (**Table 5**).

## Discussion

To our knowledge this is the first study that has validated ICD-9 related to the most frequent cardiovascular diseases, namely myocardial infarction, heart failure, and atrial fibrillation, using a large administrative database in the Italian population. Findings from the present study indicate that the assessed ICD-9 codes are highly predictive of myocardial infarction, atrial fibrillation and heart failure. Consequently, the Umbria administrative database can be used to identify the three cardiovascular diseases either as an outcome or as a covariate.

### Myocardial infarction

A systematic review published in 2014 [11] identified 30 studies that across 1984–2010 evaluated the validity of ICD-9 or ICD-10 codes related to MI and found that in more than half of the studies, sensitivity and specificity exceeded 81%, and PPV exceeded 92%; at the same time, a large number of studies reported unsatisfactory performance. The review underlined that accuracy might be influenced by the reference standard used. For example, 40% of studies used creatine kinase, lactate dehydrogenase, and aspartate transaminase enzymes as part of MONICA criteria to diagnose MI [31], whereas other studies used cardiac troponin in addition to creatine kinase according to American Heart Association (AHA) criteria, while two studies [31, 32] used the Joint European Society of Cardiology/American College of Cardiology (ESC/ACC) criteria, which is primarily based on troponin levels. The review author concluded that the introduction of cardiac troponin testing in clinical practice may have positively influenced the increase in the PPV of MI codes over time. Cardiac troponins are a more sensitive and specific indicators of myocardial injury with respect to the other enzymes, and its use is supported by many clinical and epidemiological evidence [33–35]. In our assessment, the primary requirement criterion was the increased troponin level in addition to at least one other clinical or instrumental (ECG, echocardiography) resulting in a PPV of 95%. Of the seven false positive cases, five (two case with missing troponin data and three with normal troponin value (single measurement of troponin) had evidence of occlusion or severe stenosis of the coronary vessels that required treatment with PTCA or eluting stents (DES). Two other cases had missing troponin data but reported symptoms (retrosternal chest pain radiating to the left arm and the neck (n = 1), epigastralgia associated with dyspnoea and sweating (n = 1)) and signs in the ECG (3 mm ST segment elevation in V1 and V2 with reciprocal changes in D2 and D n3 (n = 1); complete bundle branch block with subsequent regression, followed by antero-lateral reciprocal changes and subsequent death due to cardiac arrest (n = 1). Myocardial infarction can well be confirmed in these cases and if we had used broader criteria we could have obtained a PPV of 99%.

### Heart failure

Regarding heart failure, three reviews systematically summarized the results of the primary studies that validated ICD codes related to this disease [10, 28, 29]. The reviews differed in terms of inclusion criteria and obtained different results. Quach et al [28] identified 25 studies published between 1992 and 2008 that used 70 different ICD codes for defining HF. Accuracy measures were highly variable among the 21 studies validating hospital discharge abstract data, with sensitivity widely varying, ranging from 29% to 89% and PPV ranging from 12% to 100%. Conversely, Saczynski et al [29] identified 35 studies in most of which the PPV was higher than 90%. These higher accuracy measures for HF could be explained by a higher baseline risk of the populations that resulted to be older, especially in the North American databases. The most recent review by McCormick et al [10] identified 19 studies published from1999–2009, and confirmed a high specificity (≥95%) in all studies and a PPV ≥87%, but the authors concluded that administrative databases are less-than-optimal to identify HF cases, since values of sensitivities were low (≥69% in ≥50% of studies). In general, the studies used different types of reference standards including patient self-report, chart reviews by either clinicians or non-clinicians, two distinct disease registries, a single measurement of left ventricular ejection fraction, and the application of several sets of standard diagnostic criteria including the Framingham, Carlson, and European Society of Cardiology. In addition, the studies provided different algorithms to validate their criteria to adjudicate the diagnosis of HF [10].

These include the use of diagnosis in secondary position, outpatient database, the use of prescription claims of drugs such as angiotensin converting enzyme (ACE) inhibitor, angiotensin-II receptor antagonist, loop diuretic, or digoxin, and laboratory data such as BNP. Despite it is not specific to HF, Alqaisi et al [36] suggest that elevated values of BNP (≥ 200 pg/ml) in addition to any hospital discharge diagnosis + at least 2 HF outpatient encounters, improves the ability of ICD-9 codes to identify HF subjects. Our algorithm was based on a combination of signs and symptoms with at least one other conditions that includes abnormality in the ECG, BNP concentration ≥35 pg/mL and/or NT-proBNP ≥125 pg/mL, or echocardiography abnormalities. These components were derived from the algorithm proposed by the European Society of Cardiology to diagnose HF in the non-acute setting and in the acute setting [24]. As the ICD-9 codes do not distinguish between acute and non-acute settings, we combined the clinical presentation of both settings. The algorithm we used was not proposed by any study included in the systematic reviews but in our setting it provided a high sensitivity and PPV accuracy measures.

### Atrial fibrillation/flutter

A systematic review included 16 studies that validated algorithms to identify AF from administrative databases [26]. Methods used to identify AF varied across the studies and included the use of inpatient and/or outpatient data, with or without the use of ECG as an important element to validate the data, and the position of the diagnosis. Most studies used code 427.31 (atrial fibrillation), whereas four studies explicitly included atrial flutter (ICD-9 code 427.32). In terms of accuracy measures, while sensitivity ranged from 57% to 95%, the PPVs from 14 of the most relevant and comparable studies ranged from 70% to 96%. The PPV resulted lower for incident than for prevalent AF and was affected by characteristics of the algorithm, the database, and the validation criteria.

### Strengths and limitations

This study evaluated the validity of the ICD-9-CM codes for cardiovascular diseases using the administrative database of the Umbria Region. For this purpose, we reviewed original source documentation available in medical charts to adjudicate cases of cardiovascular disease.

Our study was based on a pre-published protocol, and no deviation from protocol occurred during study development.

We followed recommended guidelines based on the STARD 2015 [30] for the accurate reporting of investigations of diagnostic studies. Hence, we used detailed and explicit validation criteria, as well as duplicate and independent processes for medical chart review, data abstraction and analysis.

One limitation of our study is that we did not evaluate the accuracy of ICD-9 codes in secondary position. Another limitation of the present study concerns generalizability. In general, results that originate from a healthcare database are immediately applicable only to the setting in which such database has been validated, due to obvious differences that may exist with respect to demographics, ethnicity, disease prevalence, and standards of care, among different populations. In Italy, healthcare provision and management of healthcare databases are regulated at a national level, and therefore we speculate that results from our database, nonetheless, can be generalized to other regions in this country, also because we do not expect major differences in demographics and disease prevalence. However, replication of our validation study in other national settings may allow to explore and refine possible differences in accuracy results, and explore potential factors that might explain heterogeneity.

## Conclusion

In administrative databases, the diagnosis of a given disease is associated with a specific ICD code. Despite some limitations, the ICD code is an important tool designed to map health conditions to corresponding general disease categories, along with specific variations. These codes have the advantage of being widely available and require a much lower effort and cost than consulting medical charts [4]. Recently, the Regional Health Authority of Umbria has started a research activity regarding case definitions of several diseases [16, 17, 37, 38], and has validated ICD-9 codes for several oncological diseases [18–21]. Considering the importance of validation processes in public health, in the present study we evaluated for the first time the validity of diagnoses related to ICD-9 codes for important cardiovascular diseases namely AMI, AF/flutter, and HF, using the Regional Health database of Umbria. The case ascertainment for AMI, AF and flutter, and HF, was based on the criteria stated in the best available evidence and showed a high level of all diagnostic accuracy measures (≥90%). The healthcare administrative database of Umbria, validated for these diseases, can be confidently used for epidemiological, outcome, and health services research.

## Supporting information

S1 Table STARD-2015-Checklist. (DOCX)

S1 Dataset Minimal anonymized dataset. (PDF)
