# Supplementary material for: A diagnostic accuracy study validating cardiovascular ICD-9-CM codes in healthcare administrative databases. The Umbria Data-Value Project
Source: PLoS One. 2019 Jul 8;14(7):e0218919. doi: 10.1371/journal.pone.0218919 (PMC6613689; doi:10.1371/journal.pone.0218919)
Supplement: S1 Dataset — (PDF) [file pone.0218919.s002.pdf]

Myocardial Infarction

Number Clinical Chart	Hospital	Gender	Admission date	Discharge date	Patient's age on admission	Primary Diagnosis (ICD-9)	Symptoms Myocardial Infarction	High Troponin	ECG abnormal	Echocardiogram abnormal	Arteriography abnormal	Validation
Chart_1	Hospital_1	M	Jan-12	Jan-12	76	41011	1	1	1	1	1	1
Chart_2	Hospital_1	F	Jan-12	Feb-12	58	41011	1	1	1	1	1	1
Chart_3	Hospital_1	M	Jan-12	Feb-12	77	41071	2	1	1	1	1	1
Chart_4	Hospital_1	M	Feb-12	Feb-12	69	41071	1	1	1	0	2	1
Chart_5	Hospital_1	M	Feb-12	Feb-12	87	41041	1	1	1	1	1	1
Chart_6	Hospital_1	M	Mar-12	Mar-12	67	41041	1	1	1	1	1	1
Chart_7	Hospital_1	M	Mar-12	Apr-12	53	41071	1	1	1	1	1	1
Chart_8	Hospital_1	M	Apr-12	Apr-12	70	41041	1	1	1	1	1	1
Chart_9	Hospital_1	M	Apr-12	Apr-12	48	41011	1	1	1	1	1	1
Chart_10	Hospital_1	M	Apr-12	May-12	65	41011	1	1	1	1	2	1
Chart_11	Hospital_1	F	May-12	May-12	68	41071	1	1	0	0	1	1
Chart_12	Hospital_1	M	May-12	May-12	53	41011	1	1	1	1	2	1
Chart_13	Hospital_1	F	Jun-12	Jun-12	77	41011	1	1	1	1	1	1
Chart_14	Hospital_1	M	Jun-12	Jun-12	51	41041	1	1	1	1	2	1
Chart_15	Hospital_1	M	Jul-12	Jul-12	48	41041	1	1	1	1	1	1
Chart_16	Hospital_1	M	Aug-12	Aug-12	62	41071	1	1	0	0	1	1
Chart_17	Hospital_1	M	Oct-12	Oct-12	84	41071	1	1	0	0	1	1
Chart_18	Hospital_1	M	Oct-12	Oct-12	76	41091	1	1	0	0	2	1
Chart_19	Hospital_1	F	Nov-12	Nov-12	89	41091	2	2	0	2	2	0
Chart_20	Hospital_1	M	Jan-13	Jan-13	67	41011	1	1	1	0	2	1
Chart_21	Hospital_1	M	Jan-13	Jan-13	50	41011	1	2	1	2	1	0
Chart_22	Hospital_1	F	Jan-13	Feb-13	73	41041	1	1	1	1	1	1
Chart_23	Hospital_1	M	Feb-13	Feb-13	84	41071	0	1	1	1	1	1
Chart_24	Hospital_1	F	Feb-13	Feb-13	66	41041	1	1	1	1	1	1
Chart_25	Hospital_1	F	Apr-13	Apr-13	76	41041	1	1	1	1	1	1
Chart_26	Hospital_1	M	Apr-13	May-13	69	41071	1	1	1	1	1	1
Chart_27	Hospital_1	F	May-13	May-13	64	41071	1	1	1	1	1	1
Chart_28	Hospital_1	F	May-13	Jun-13	74	41011	1	1	1	1	2	1
Chart_29	Hospital_1	M	Jun-13	Jun-13	65	41041	1	1	1	1	1	1
Chart_30	Hospital_1	M	Jul-13	Jul-13	72	41041	1	0	1	1	1	0
Chart_31	Hospital_1	M	Sep-13	Oct-13	72	41001	1	1	1	1	2	1
Chart_32	Hospital_1	M	Oct-13	Nov-13	70	41041	1	1	1	1	2	1
Chart_33	Hospital_1	F	Oct-13	Nov-13	57	41041	1	1	1	1	1	1

Number Clinical Chart	Hospital	Gender	Admission date	Discharge date	Patient's age on admission	Primary Diagnosis (ICD-9)	Symptoms Myocardial Infarction	High Troponin	ECG abnormal	Echocardiogram abnormal	Arteriography abnormal	Validation
Chart_34	Hospital_1	M	Nov-13	Nov-13	49	41041	1	1	1	1	1	1
Chart_35	Hospital_1	M	Nov-13	Nov-13	49	41011	1	1	1	1	1	1
Chart_36	Hospital_1	F	Dec-13	Dec-13	51	41051	1	1	1	0	1	1
Chart_37	Hospital_1	M	Dec-13	Dec-13	88	41071	1	1	1	1	2	1
Chart_38	Hospital_1	M	Dec-13	Dec-13	104	41071	1	1	0	1	2	1
Chart_39	Hospital_1	F	Jan-14	Jan-14	56	41041	1	1	1	0	1	1
Chart_40	Hospital_1	M	Feb-14	Feb-14	53	41071	1	1	1	0	1	1
Chart_41	Hospital_1	F	Mar-14	Mar-14	82	41011	1	1	1	1	1	1
Chart_42	Hospital_1	M	Mar-14	Mar-14	70	41071	1	1	1	0	1	1
Chart_43	Hospital_1	M	Mar-14	Apr-14	82	41071	1	1	1	1	1	1
Chart_44	Hospital_1	F	Apr-14	May-14	67	41011	1	1	1	1	1	1
Chart_45	Hospital_1	M	Apr-14	Apr-14	48	41011	1	1	1	1	1	1
Chart_46	Hospital_1	M	May-14	Jun-14	87	41071	1	1	1	0	1	1
Chart_47	Hospital_1	M	Aug-14	Aug-14	50	41031	1	1	1	1	1	1
Chart_48	Hospital_1	M	Oct-14	Nov-14	74	41011	1	1	1	1	1	1
Chart_49	Hospital_1	F	Oct-14	Oct-14	70	41011	0	0	1	1	1	0
Chart_50	Hospital_1	F	Dec-14	Dec-14	61	41011	1	1	1	0	1	1
Chart_51	Hospital_1	M	Dec-14	Dec-14	65	41041	1	1	1	1	1	1
Chart_52	Hospital_2	F	Jan-12	Jan-12	71	41021	1	1	1	1	1	1
Chart_53	Hospital_2	F	Mar-12	Mar-12	64	41021	1	1	1	1	1	1
Chart_54	Hospital_2	M	Mar-12	Mar-12	74	41011	1	1	1	0	2	1
Chart_55	Hospital_2	M	Aug-12	Aug-12	87	41071	1	1	1	0	1	1
Chart_56	Hospital_2	M	Sep-12	Sep-12	62	41021	1	1	1	0	0	1
Chart_57	Hospital_2	M	Sep-12	Sep-12	75	41021	0	0	2	1	1	0
Chart_58	Hospital_2	M	Sep-12	Sep-12	74	41011	1	1	1	0	1	1
Chart_59	Hospital_2	M	Oct-12	Oct-12	64	41011	1	1	1	1	1	1
Chart_60	Hospital_2	M	Nov-12	Nov-12	84	41091	1	1	1	1	1	1
Chart_61	Hospital_2	F	May-13	May-13	92	41071	1	1	1	0	1	1
Chart_62	Hospital_2	M	May-13	May-13	78	41011	1	1	1	1	1	1
Chart_63	Hospital_2	M	Jul-13	Jul-13	69	41011	1	1	1	1	1	1
Chart_64	Hospital_2	F	Jul-13	Jul-13	83	41091	1	1	1	1	1	1
Chart_65	Hospital_2	M	Jul-13	Jul-13	89	41011	1	1	1	1	2	1
Chart_66	Hospital_2	M	Aug-13	Aug-13	56	41011	1	1	0	0	1	1
Chart_67	Hospital_2	F	Oct-13	Oct-13	57	41011	1	1	1	1	1	1
Chart_68	Hospital_2	M	Oct-13	Oct-13	83	41011	1	1	1	1	2	1

Number Clinical Chart	Hospital	Gender	Admission date	Discharge date	Patient's age on admission	Primary Diagnosis (ICD-9)	Symptoms Myocardial Infarction	High Troponin	ECG abnormal	Echocardiogram abnormal	Arteriography abnormal	Validation
Chart_69	Hospital_2	F	Oct-13	Oct-13	89	41071	1	1	1	1	2	1
Chart_70	Hospital_2	M	Oct-13	Nov-13	--	41071	n.a.	n.a.	n.a.	n.a.	n.a.	n.a.
Chart_71	Hospital_2	M	Dec-13	Dec-13	77	41021	1	1	1	1	1	1
Chart_72	Hospital_2	F	Jan-14	Feb-14	89	41000	1	1	0	0	2	1
Chart_73	Hospital_2	M	Jan-14	Feb-14	80	41071	1	1	1	0	1	1
Chart_74	Hospital_2	M	May-14	May-14	45	41021	1	1	1	1	1	1
Chart_75	Hospital_2	F	Jun-14	Jun-14	81	41072	1	1	1	1	2	1
Chart_76	Hospital_2	F	Jul-14	Jul-14	74	41071	1	1	0	0	1	1
Chart_77	Hospital_2	M	Sep-14	Sep-14	38	41011	1	1	1	1	1	1
Chart_78	Hospital_2	M	Nov-14	Nov-14	93	41041	1	1	1	1	2	1
Chart_79	Hospital_2	M	Nov-14	Nov-14	47	41011	1	1	1	1	1	1
Chart_80	Hospital_2	M	Nov-14	Nov-14	72	41071	1	1	1	1	1	1
Chart_81	Hospital_2	M	Nov-14	Dec-14	--	41071	n.a.	n.a.	n.a.	n.a.	n.a.	n.a.
Chart_82	Hospital_2	M	Dec-14	Dec-14	78	41071	1	1	1	0	1	1
Chart_83	Hospital_3	M	Jan-12	Jan-12	92	41041	2	1	1	0	2	1
Chart_84	Hospital_3	F	Apr-12	May-12	77	41071	1	1	0	0	1	1
Chart_85	Hospital_3	M	Jan-13	Jan-13	76	41071	1	2	0	0	1	0
Chart_86	Hospital_3	F	Mar-13	Mar-13	92	41001	1	1	1	1	2	1
Chart_87	Hospital_3	M	Jun-13	Jun-13	81	41071	1	1	0	0	2	1
Chart_88	Hospital_3	M	Jun-13	Jun-13	74	41071	1	1	1	0	2	1
Chart_89	Hospital_3	F	Nov-13	Nov-13	84	41071	0	1	1	1	2	1
Chart_90	Hospital_3	F	Feb-14	Feb-14	90	41071	1	1	1	1	2	1
Chart_91	Hospital_3	F	May-14	May-14	88	41090	1	1	0	0	2	1
Chart_92	Hospital_4	M	Apr-13	May-13	55	41071	1	1	1	0	1	1
Chart_93	Hospital_4	M	Sep-12	Sep-12	80	41011	1	1	1	1	2	1
Chart_94	Hospital_4	M	Oct-13	Nov-13	75	41091	1	1	2	1	1	1
Chart_95	Hospital_4	F	May-13	Jul-13	87	41091	1	1	1	0	2	1
Chart_96	Hospital_4	M	Mar-13	Mar-13	66	41041	1	1	1	1	1	1
Chart_97	Hospital_4	F	Mar-13	Mar-13	74	41011	1	1	1	1	2	1
Chart_98	Hospital_4	M	Apr-12	Apr-12	52	41071	1	1	0	0	2	1
Chart_99	Hospital_4	F	Jul-14	Jul-14	87	41091	1	2	1	1	2	0
Chart_100	Hospital_4	M	Mar-12	Mar-12	77	41071	1	1	1	0	2	1
Chart_101	Hospital_5	F	Jan-12	Jan-12	76	41071	1	1	1	0	1	1
Chart_102	Hospital_5	M	Apr-12	Apr-12	55	41071	1	1	1	1	1	1
Chart_103	Hospital_5	M	Apr-12	May-12	72	41072	1	1	1	0	1	1

Number Clinical Chart	Hospital	Gender	Admission date	Discharge date	Patient's age on admission	Primary Diagnosis (ICD-9)	Symptoms Myocardial Infarction	High Troponin	ECG abnormal	Echocardiogram abnormal	Arteriography abnormal	Validation
Chart_104	Hospital_5	M	May-12	May-12	74	41071	1	1	0	1	1	1
Chart_105	Hospital_5	M	Jun-12	Jun-12	68	41071	1	1	1	1	1	1
Chart_106	Hospital_5	M	Oct-12	Nov-12	76	41011	1	1	1	1	1	1
Chart_107	Hospital_5	F	Mar-13	Mar-13	88	41081	1	1	2	0	1	1
Chart_108	Hospital_5	M	May-13	Jun-13	64	41041	1	1	1	1	1	1
Chart_109	Hospital_5	M	Sep-13	Sep-13	55	41041	1	1	1	0	1	1
Chart_110	Hospital_5	M	Dec-13	Jan-14	78	41021	1	1	1	1	1	1
Chart_111	Hospital_5	M	Jan-14	Jan-14	87	41071	1	1	1	1	1	1
Chart_112	Hospital_5	F	Mar-14	Mar-14	85	41041	1	1	1	1	2	1
Chart_113	Hospital_5	M	May-14	Jun-14	58	41071	1	1	1	0	1	1
Chart_114	Hospital_5	M	Jul-14	Jul-14	59	41041	1	1	1	1	1	1
Chart_115	Hospital_5	F	Sep-14	Sep-14	69	41071	1	1	1	1	0	1
Chart_116	Hospital_5	M	Nov-14	Nov-14	59	41011	1	1	1	1	1	1
Chart_117	Hospital_5	M	Dec-14	Dec-14	44	41071	1	1	1	1	1	1
Chart_118	Hospital_6	M	Jan-12	Jan-12	73	41021	1	1	1	1	2	1
Chart_119	Hospital_6	F	Feb-12	Mar-12	81	41071	1	1	1	0	2	1
Chart_120	Hospital_6	M	Nov-12	Nov-12	84	41071	1	1	0	1	2	1
Chart_121	Hospital_6	M	Nov-12	Dec-12	79	41071	1	1	1	0	2	1
Chart_122	Hospital_6	F	Dec-12	Jan-13	97	41071	1	1	1	0	2	1
Chart_123	Hospital_6	M	Feb-14	Mar-14	76	41071	1	1	1	1	2	1
Chart_124	Hospital_7	M	Jan-12	Jan-12	74	41071	1	1	1	1	2	1
Chart_125	Hospital_7	F	May-12	May-12	82	41071	1	1	1	1	2	1
Chart_126	Hospital_7	F	Aug-13	Aug-13	66	41071	1	1	0	1	2	1
Chart_127	Hospital_7	M	Sep-13	Sep-13	47	41071	1	1	0	0	2	1
Chart_128	Hospital_7	M	Oct-13	Oct-13	80	41071	1	1	0	0	2	1
Chart_129	Hospital_7	F	Nov-13	Nov-13	65	41071	1	1	1	0	2	1
Chart_130	Hospital_7	F	Dec-14	Dec-14	80	41071	0	1	1	1	2	1

Legend: 0=no; 1=yes; 2=not reported; n.a.=clinical chart not available

Atrial fibrillation / flutter

Number Clinical Chart	Hospital	Gender	Admission date	Discharge date	Patient's age on admission	Primary Diagnosis (ICD-9)	ECG abnormal with presence of AF/Flutter	Validation
chart_1	Hospital_1	M	Jan-12	Jan-12	73	42732	1	1
chart_2	Hospital_1	M	Feb-12	Feb-12	61	42732	1	1
chart_3	Hospital_1	M	Feb-12	Feb-12	65	42731	1	1
chart_4	Hospital_1	F	Mar-12	Mar-12	83	42731	1	1
chart_5	Hospital_1	M	Apr-12	Apr-12	72	42732	1	1
chart_6	Hospital_1	M	May-12	May-12	49	42731	1	1
chart_7	Hospital_1	F	May-12	May-12	72	42731	1	1
chart_8	Hospital_1	M	Jun-12	Jun-12	77	42731	1	1
chart_9	Hospital_1	F	Jun-12	Jul-12	83	42732	1	1
chart_10	Hospital_1	M	Jul-12	Jul-12	--	42731	n.a.	n.a.
chart_11	Hospital_1	F	Aug-12	Aug-12	79	42732	1	1
chart_12	Hospital_1	M	Nov-12	Nov-12	55	42731	1	1
chart_13	Hospital_1	M	Jan-13	Feb-13	86	42731	1	1
chart_14	Hospital_1	M	Feb-13	Feb-13	72	42731	1	1
chart_15	Hospital_1	F	Apr-13	Apr-13	53	42731	1	1
chart_16	Hospital_1	M	May-13	May-13	38	42731	1	1
chart_17	Hospital_1	F	May-13	May-13	52	42731	1	1
chart_18	Hospital_1	M	Jun-13	Jun-13	62	42731	1	1
chart_19	Hospital_1	F	Jun-13	Jul-13	83	42731	1	1
chart_20	Hospital_1	F	Jul-13	Jul-13	91	42731	1	1
chart_21	Hospital_1	M	Jul-13	Jul-13	54	42731	1	1
chart_22	Hospital_1	M	Aug-13	Aug-13	83	42731	1	1
chart_23	Hospital_1	M	Sep-13	Sep-13	47	42731	1	1
chart_24	Hospital_1	F	Oct-13	Oct-13	73	42731	1	1
chart_25	Hospital_1	M	Nov-13	Dec-13	--	42732	n.a.	n.a.
chart_26	Hospital_1	F	Dec-13	Dec-13	66	42731	1	1
chart_27	Hospital_1	F	Dec-13	Dec-13	61	42731	1	1
chart_28	Hospital_1	F	Dec-13	Dec-13	89	42731	1	1
chart_29	Hospital_1	F	Dec-13	Dec-13	78	42731	1	1
chart_30	Hospital_1	F	Jan-14	Jan-14	80	42731	1	1
chart_31	Hospital_1	M	Feb-14	Feb-14	90	42731	1	1
chart_32	Hospital_1	F	Feb-14	Feb-14	83	42731	1	1

Number Clinical Chart	Hospital	Gender	Admission date	Discharge date	Patient's age on admission	Primary Diagnosis (ICD-9)	ECG abnormal with presence of AF/Flutter	Validation
chart_33	Hospital_1	F	Feb-14	Feb-14	64	42732	1	1
chart_34	Hospital_1	F	Mar-14	Mar-14	71	42731	1	1
chart_35	Hospital_1	M	Mar-14	Mar-14	80	42731	1	1
chart_36	Hospital_1	F	May-14	May-14	67	42732	0	0
chart_37	Hospital_1	M	Jun-14	Jun-14	76	42732	1	1
chart_38	Hospital_1	M	Jun-14	Jun-14	83	42731	1	1
chart_39	Hospital_1	M	Jun-14	Jul-14	92	42731	1	1
chart_40	Hospital_1	M	Jul-14	Jul-14	67	42731	1	1
chart_41	Hospital_1	F	Oct-14	Oct-14	93	42731	1	1
chart_42	Hospital_1	F	Oct-14	Oct-14	77	42731	1	1
chart_43	Hospital_1	M	Nov-14	Nov-14	84	42732	1	1
chart_44	Hospital_2	F	Dec-11	Jan-12	87	42731	1	1
chart_45	Hospital_2	M	Jan-12	Jan-12	43	42731	1	1
chart_46	Hospital_2	F	Jan-12	Jan-12	81	42731	1	1
chart_47	Hospital_2	M	Feb-12	Feb-12	92	42731	1	1
chart_48	Hospital_2	F	Feb-12	Mar-12	88	42731	1	1
chart_49	Hospital_2	F	Feb-12	Mar-12	72	42731	1	1
chart_50	Hospital_2	M	Apr-12	Apr-12	76	42731	1	1
chart_51	Hospital_2	M	Apr-12	Apr-12	54	42731	1	1
chart_52	Hospital_2	M	Apr-12	Apr-12	63	42731	1	1
chart_53	Hospital_2	F	May-12	May-12	73	42731	1	1
chart_54	Hospital_2	M	Jun-12	Jun-12	77	42731	1	1
chart_55	Hospital_2	F	Jun-12	Jun-12	78	42731	1	1
chart_56	Hospital_2	M	Sep-12	Sep-12	82	42731	1	1
chart_57	Hospital_2	M	Sep-12	Oct-12	85	42731	1	1
chart_58	Hospital_2	M	Nov-12	Nov-12	77	42732	1	1
chart_59	Hospital_2	F	Nov-12	Nov-12	55	42731	1	1
chart_60	Hospital_2	F	Nov-12	Dec-12	77	42731	1	1
chart_61	Hospital_2	M	Dec-12	Dec-12	54	42731	1	1
chart_62	Hospital_2	F	Jan-13	Jan-13	85	42731	2	0
chart_63	Hospital_2	F	Jan-13	Jan-13	63	42731	1	1
chart_64	Hospital_2	F	Jan-13	Jan-13	89	42731	1	1
chart_65	Hospital_2	M	Jan-13	Jan-13	62	42731	1	1
chart_66	Hospital_2	M	Feb-13	Feb-13	--	42731	n.a.	n.a.
chart_67	Hospital_2	M	Mar-13	Mar-13	77	42732	1	1

Number Clinical Chart	Hospital	Gender	Admission date	Discharge date	Patient's age on admission	Primary Diagnosis (ICD-9)	ECG abnormal with presence of AF/Flutter	Validation
chart_68	Hospital_2	F	Mar-13	Mar-13	93	42731	1	1
chart_69	Hospital_2	M	Apr-13	Apr-13	68	42732	1	1
chart_70	Hospital_2	F	Apr-13	May-13	76	42732	1	1
chart_71	Hospital_2	F	May-13	May-13	80	42732	1	1
chart_72	Hospital_2	F	Jul-13	Jul-13	71	42732	1	1
chart_73	Hospital_2	F	Nov-13	Nov-13	71	42731	1	1
chart_74	Hospital_2	F	Dec-13	Dec-13	56	42731	1	1
chart_75	Hospital_2	F	Dec-13	Dec-13	66	42731	0	0
chart_76	Hospital_2	F	Dec-13	Dec-13	76	42731	1	1
chart_77	Hospital_2	M	Jan-14	Jan-14	70	42731	1	1
chart_78	Hospital_2	M	Jan-14	Jan-14	71	42731	1	1
chart_79	Hospital_2	M	Jan-14	Jan-14	76	42731	1	1
chart_80	Hospital_2	F	Mar-14	Mar-14	89	42731	1	1
chart_81	Hospital_2	F	Mar-14	Mar-14	75	42731	1	1
chart_82	Hospital_2	F	Apr-14	Apr-14	96	42731	1	1
chart_83	Hospital_2	M	Jun-14	Jun-14	74	42731	1	1
chart_84	Hospital_2	F	Jun-14	Jun-14	82	42732	1	1
chart_85	Hospital_2	M	Aug-14	Aug-14	84	42731	1	1
chart_86	Hospital_2	M	Oct-14	Oct-14	76	42731	1	1
chart_87	Hospital_2	F	Nov-14	Nov-14	95	42731	1	1
chart_88	Hospital_2	F	Nov-14	Nov-14	78	42731	1	1
chart_89	Hospital_2	M	Dec-14	Dec-14	82	42731	1	1
chart_90	Hospital_3	M	Sep-12	Sep-12	67	42731	1	1
chart_91	Hospital_3	M	Dec-12	Dec-12	64	42731	1	1
chart_92	Hospital_3	M	Apr-13	May-13	77	42731	1	1
chart_93	Hospital_3	M	May-13	May-13	61	42731	1	1
chart_94	Hospital_3	F	Jun-13	Jun-13	77	42731	0	0
chart_95	Hospital_3	M	Apr-14	Apr-14	71	42731	1	1
chart_96	Hospital_4	M	Jan-12	Jan-12	64	42732	1	1
chart_97	Hospital_4	M	Jan-12	Jan-12	69	42731	1	1
chart_98	Hospital_4	M	Jun-12	Jun-12	64	42732	1	1
chart_99	Hospital_4	F	Jul-12	Jul-12	84	42731	1	1
chart_100	Hospital_4	M	Dec-13	Dec-13	77	42731	1	1
chart_101	Hospital_4	M	May-14	May-14	73	42731	1	1
chart_102	Hospital_4	F	Jun-14	Jun-14	79	42731	1	1

Number Clinical Chart	Hospital	Gender	Admission date	Discharge date	Patient's age on admission	Primary Diagnosis (ICD-9)	ECG abnormal with presence of AF/Flutter	Validation
chart_103	Hospital_4	M	Jun-14	Jun-14	67	42731	1	1
chart_104	Hospital_5	F	Dec-11	Jan-12	87	42731	1	1
chart_105	Hospital_5	F	Feb-12	Mar-12	92	42731	1	1
chart_106	Hospital_5	F	Jun-12	Jun-12	87	42731	1	1
chart_107	Hospital_5	F	Aug-12	Aug-12	82	42731	1	1
chart_108	Hospital_5	F	Aug-12	Aug-12	89	42731	1	1
chart_109	Hospital_5	F	Jul-13	Jul-13	90	42731	1	1
chart_110	Hospital_5	M	Oct-13	Oct-13	81	42732	1	1
chart_111	Hospital_5	M	May-14	May-14	69	42732	1	1
chart_112	Hospital_5	M	May-14	May-14	79	42731	1	1
chart_113	Hospital_6	M	Sep-12	Sep-12	83	42731	1	1
chart_114	Hospital_6	F	Nov-12	Nov-12	82	42731	1	1
chart_115	Hospital_6	M	Jan-13	Jan-13	75	42731	1	1
chart_116	Hospital_6	M	Jan-13	Jan-13	62	42731	1	1
chart_117	Hospital_6	M	Sep-13	Sep-13	56	42731	1	1
chart_118	Hospital_6	F	Oct-13	Oct-13	52	42731	1	1
chart_119	Hospital_6	M	Oct-13	Oct-13	70	42731	1	1
chart_120	Hospital_6	F	Jan-14	Feb-14	87	42731	1	1
chart_121	Hospital_6	M	Jan-14	Feb-14	71	42731	1	1
chart_122	Hospital_6	F	Mar-14	Mar-14	80	42731	1	1
chart_123	Hospital_6	M	Apr-14	Apr-14	71	42731	1	1
chart_124	Hospital_6	M	Jun-14	Jun-14	81	42731	1	1
chart_125	Hospital_6	M	Jul-14	Jul-14	80	42731	1	1
chart_126	Hospital_6	M	Oct-14	Oct-14	68	42731	1	1
chart_127	Hospital_6	F	Dec-14	Dec-14	88	42731	1	1
chart_128	Hospital_6	M	Dec-14	Dec-14	68	42731	1	1
chart_129	Hospital_7	F	Oct-12	Oct-12	98	42731	1	1
chart_130	Hospital_7	M	Nov-12	Nov-12	68	42731	1	1

Legend: 0=no; 1=yes; 2=not reported; n.a.=clinical chart not available

Heart failure

Number Clinical Chart	Hospital	Gender	Admission date	Discharge date	Patient's age on admission	Primary Diagnosis (ICD-9)	Symptoms of heart failure	ECG abnormal	BNP high	Echocardiogram abnormal	Validation
Chart_1	Hospital_1	M	Feb-12	Feb-12	71	4280	1	1	1	1	1
Chart_2	Hospital_1	M	Feb-12	Feb-12	82	4281	1	1	1	1	1
Chart_3	Hospital_1	F	Mar-12	Apr-12	82	4289	1	0	2	1	1
Chart_4	Hospital_1	F	Apr-12	Apr-12	69	4281	1	1	1	1	1
Chart_5	Hospital_1	F	Apr-12	Apr-12	75	4281	1	1	1	1	1
Chart_6	Hospital_1	F	Jun-12	Jul-12	71	4280	1	0	1	1	1
Chart_7	Hospital_1	M	Aug-12	Sep-12	73	4281	1	0	1	1	1
Chart_8	Hospital_1	F	Oct-12	Oct-12	69	4281	1	1	1	1	1
Chart_9	Hospital_1	F	Oct-12	Oct-12	75	4280	1	1	1	1	1
Chart_10	Hospital_1	F	Dec-12	Dec-12	78	42841	1	1	1	0	1
Chart_11	Hospital_1	F	Mar-13	Mar-13	69	42833	1	1	1	1	1
Chart_12	Hospital_1	F	Apr-13	Apr-13	--	4280	n.a.	n.a.	n.a.	n.a.	n.a.
Chart_13	Hospital_1	F	May-13	May-13	79	4280	1	1	2	1	1
Chart_14	Hospital_1	F	Jun-13	Jun-13	84	4281	1	1	1	1	1
Chart_15	Hospital_1	F	Sep-13	Sep-13	77	4280	1	1	0	2	1
Chart_16	Hospital_1	M	Sep-13	Sep-13	77	4281	1	1	1	1	1
Chart_17	Hospital_1	F	Oct-13	Oct-13	89	4280	1	1	1	1	1
Chart_18	Hospital_1	M	Nov-13	Nov-13	73	4280	1	1	1	1	1
Chart_19	Hospital_1	F	Nov-13	Dec-13	86	4281	1	0	1	1	1
Chart_20	Hospital_1	M	Dec-13	Dec-13	78	4280	1	1	2	2	1
Chart_21	Hospital_1	M	Dec-13	Dec-13	80	4280	1	1	1	1	1
Chart_22	Hospital_1	M	Jan-14	Jan-14	59	4280	1	1	1	1	1
Chart_23	Hospital_1	F	Jan-14	Jan-14	70	4281	1	1	1	1	1
Chart_24	Hospital_1	F	Jan-14	Jan-14	91	4280	1	1	1	1	1
Chart_25	Hospital_1	M	Jan-14	Feb-14	97	4280	1	1	1	2	1
Chart_26	Hospital_1	M	Feb-14	Mar-14	83	4280	1	1	1	1	1
Chart_27	Hospital_1	F	Feb-14	Mar-14	62	4281	1	1	1	1	1
Chart_28	Hospital_1	M	Mar-14	Mar-14	88	42823	1	1	1	2	1
Chart_29	Hospital_1	F	Mar-14	Apr-14	89	4280	1	0	1	1	1
Chart_30	Hospital_1	F	Apr-14	Apr-14	91	4281	1	1	0	0	1
Chart_31	Hospital_1	F	May-14	May-14	52	4281	1	1	2	2	1
Chart_32	Hospital_1	M	Jul-14	Jul-14	89	4281	1	1	1	2	1
Chart_33	Hospital_1	F	Jul-14	Jul-14	83	4281	1	1	1	1	1

Number Clinical Chart	Hospital	Gender	Admission date	Discharge date	Patient's age on admission	Primary Diagnosis (ICD-9)	Symptoms of heart failure	ECG abnormal	BNP high	Echocardiogram abnormal	Validation
Chart_34	Hospital_1	F	Aug-14	Aug-14	71	4289	1	1	2	0	1
Chart_35	Hospital_1	F	Aug-14	Sep-14	--	4280	n.a.	n.a.	n.a.	n.a.	n.a.
Chart_36	Hospital_1	F	Aug-14	Aug-14	86	42823	1	1	1	1	1
Chart_37	Hospital_1	M	Sep-14	Sep-14	87	4281	1	1	1	1	1
Chart_38	Hospital_2	F	Feb-12	Feb-12	92	4280	1	1	2	2	1
Chart_39	Hospital_2	M	Feb-12	Feb-12	76	4280	1	0	2	1	1
Chart_40	Hospital_2	F	Feb-12	Mar-12	92	4280	1	1	2	2	1
Chart_41	Hospital_2	F	Feb-12	Mar-12	80	4280	1	1	2	2	1
Chart_42	Hospital_2	M	Feb-12	Feb-12	83	4280	1	1	2	2	1
Chart_43	Hospital_2	F	Mar-12	Mar-12	66	4280	0	1	2	2	0
Chart_44	Hospital_2	F	Apr-12	Apr-12	86	4280	1	0	2	2	0
Chart_45	Hospital_2	F	Apr-12	Apr-12	97	4280	1	0	2	2	0
Chart_46	Hospital_2	F	Apr-12	May-12	73	4280	1	1	2	1	1
Chart_47	Hospital_2	F	May-12	May-12	72	4280	1	1	2	2	1
Chart_48	Hospital_2	M	May-12	May-12	86	4280	1	1	2	2	1
Chart_49	Hospital_2	M	Jul-12	Jul-12	88	4280	1	2	2	2	0
Chart_50	Hospital_2	M	Oct-12	Oct-12	77	4280	1	1	2	1	1
Chart_51	Hospital_2	M	Oct-12	Oct-12	--	4280	n.a.	n.a.	n.a.	n.a.	n.a.
Chart_52	Hospital_2	M	Dec-12	Jan-13	70	4280	1	1	2	1	1
Chart_53	Hospital_2	F	Jan-13	Jan-13	84	4280	1	1	2	2	1
Chart_54	Hospital_2	M	Jan-13	Jan-13	91	4280	1	1	2	2	1
Chart_55	Hospital_2	M	Jan-13	Jan-13	89	4280	1	1	2	2	1
Chart_56	Hospital_2	F	Feb-13	Feb-13	86	4281	1	1	2	2	1
Chart_57	Hospital_2	F	Mar-13	Apr-13	78	4280	1	1	2	2	1
Chart_58	Hospital_2	M	Apr-13	Apr-13	93	4280	1	2	2	2	0
Chart_59	Hospital_2	F	Apr-13	Apr-13	83	4280	1	1	2	2	1
Chart_60	Hospital_2	M	May-13	Jun-13	87	4280	1	1	2	1	1
Chart_61	Hospital_2	F	Jul-13	Aug-13	85	4280	1	0	2	2	0
Chart_62	Hospital_2	F	Sep-13	Sep-13	34	4280	1	0	2	2	0
Chart_63	Hospital_2	M	Sep-13	Oct-13	91	4280	1	1	2	2	1
Chart_64	Hospital_2	F	Oct-13	Nov-13	85	4280	1	1	2	1	1
Chart_65	Hospital_2	M	Nov-13	Nov-13	82	4280	1	1	2	1	1
Chart_66	Hospital_2	M	Nov-13	Nov-13	71	4280	1	1	2	1	1
Chart_67	Hospital_2	F	Nov-13	Dec-13	92	4280	1	1	2	1	1
Chart_68	Hospital_2	M	Mar-14	Mar-14	73	4280	1	1	2	1	1

Number Clinical Chart	Hospital	Gender	Admission date	Discharge date	Patient's age on admission	Primary Diagnosis (ICD-9)	Symptoms of heart failure	ECG abnormal	BNP high	Echocardiog ram abnormal	Validation
Chart_69	Hospital_2	F	Mar-14	Mar-14	86	4280	1	1	2	2	1
Chart_70	Hospital_2	F	Nov-14	Dec-14	99	4280	1	1	2	2	1
Chart_71	Hospital_2	F	Dec-14	Dec-14	81	4280	1	1	2	2	1
Chart_72	Hospital_2	M	Dec-14	Dec-14	52	4280	1	1	2	1	1
Chart_73	Hospital_2	M	Dec-14	Dec-14	79	4280	1	1	2	2	1
Chart_74	Hospital_3	F	Mar-12	Apr-12	74	4280	1	1	2	1	1
Chart_75	Hospital_3	F	Aug-12	Aug-12	87	4281	1	2	1	2	1
Chart_76	Hospital_3	M	Dec-12	Jan-13	79	4281	1	1	2	1	1
Chart_77	Hospital_3	M	Jan-13	Jan-13	78	4281	1	1	1	1	1
Chart_78	Hospital_3	F	Jun-13	Jun-13	78	4281	1	1	1	1	1
Chart_79	Hospital_3	M	Sep-13	Sep-13	87	4281	1	1	2	2	1
Chart_80	Hospital_3	M	Nov-13	Dec-13	81	42843	1	1	1	1	1
Chart_81	Hospital_3	M	Jan-14	Feb-14	80	42843	1	1	2	1	1
Chart_82	Hospital_3	F	Mar-14	Mar-14	68	42843	1	1	1	1	1
Chart_83	Hospital_3	F	Apr-14	Apr-14	74	42843	1	1	2	1	1
Chart_84	Hospital_3	M	Jul-14	Jul-14	74	42843	1	1	1	1	1
Chart_85	Hospital_3	M	Oct-14	Oct-14	85	4280	1	1	2	2	1
Chart_86	Hospital_4	F	Feb-12	Feb-12	78	4281	1	0	0	0	0
Chart_87	Hospital_4	M	May-12	May-12	78	4280	1	1	1	2	1
Chart_88	Hospital_4	M	Sep-12	Oct-12	77	4281	1	1	2	1	1
Chart_89	Hospital_4	M	Oct-12	Oct-12	80	4280	1	1	1	1	1
Chart_90	Hospital_4	F	Nov-12	Nov-12	83	4280	1	0	1	2	1
Chart_91	Hospital_4	F	Apr-13	Apr-13	79	4280	1	1	1	2	1
Chart_92	Hospital_4	M	Jun-13	Jul-13	89	4280	1	1	2	1	1
Chart_93	Hospital_4	F	Dec-13	Jan-14	81	4281	1	0	2	1	1
Chart_94	Hospital_4	F	Jul-14	Jul-14	79	4281	1	1	2	1	1
Chart_95	Hospital_5	M	Dec-11	Jan-12	83	4280	1	1	2	2	1
Chart_96	Hospital_5	F	Jan-12	Jan-12	88	4281	1	1	2	1	1
Chart_97	Hospital_5	F	Mar-12	Mar-12	89	4280	1	1	2	2	1
Chart_98	Hospital_5	M	Apr-12	May-12	83	4281	1	1	2	1	1
Chart_99	Hospital_5	M	Aug-12	Aug-12	83	4280	1	2	1	1	1
Chart_100	Hospital_5	M	Nov-12	Dec-12	86	4280	1	1	2	1	1
Chart_101	Hospital_5	F	Feb-13	Feb-13	84	4280	1	1	2	0	1
Chart_102	Hospital_5	F	Feb-13	Mar-13	90	4280	1	1	2	1	1
Chart_103	Hospital_5	F	Mar-13	Mar-13	85	4280	1	1	2	1	1

Number Clinical Chart	Hospital	Gender	Admission date	Discharge date	Patient's age on admission	Primary Diagnosis (ICD-9)	Symptoms of heart failure	ECG abnormal	BNP high	Echocardiogram abnormal	Validation
Chart_104	Hospital_5	F	Mar-13	Apr-13	75	4281	1	1	2	1	1
Chart_105	Hospital_5	F	Feb-14	Feb-14	83	4280	1	1	1	2	1
Chart_106	Hospital_5	M	Jun-14	Jun-14	64	4280	0	1	2	1	1
Chart_107	Hospital_5	M	Aug-14	Aug-14	80	4280	1	1	2	1	1
Chart_108	Hospital_5	F	Oct-14	Nov-14	80	4280	1	1	1	0	1
Chart_109	Hospital_5	M	Nov-14	Nov-14	77	4280	1	0	2	1	1
Chart_110	Hospital_6	F	Aug-12	Aug-12	82	4281	1	0	1	0	1
Chart_111	Hospital_6	M	Aug-12	Sep-12	88	4280	1	1	1	1	1
Chart_112	Hospital_6	M	Jan-13	Jan-13	86	4280	1	1	1	1	1
Chart_113	Hospital_6	F	May-13	May-13	81	4280	1	1	1	0	1
Chart_114	Hospital_6	M	Jun-13	Jun-13	60	42830	1	0	2	1	1
Chart_115	Hospital_6	F	Jun-13	Jun-13	95	4280	1	1	1	0	1
Chart_116	Hospital_6	F	Dec-13	Dec-13	82	4280	1	1	1	2	1
Chart_117	Hospital_6	M	Mar-14	Apr-14	89	4280	1	1	2	2	1
Chart_118	Hospital_6	F	Oct-14	Oct-14	86	42841	1	1	2	2	1
Chart_119	Hospital_6	M	Oct-14	Oct-14	75	42823	1	1	2	1	1
Chart_120	Hospital_6	F	Dec-14	Dec-14	84	42830	1	1	1	1	1
Chart_121	Hospital_6	M	Dec-14	Dec-14	91	42841	1	0	1	1	1
Chart_122	Hospital_7	F	Jul-12	Aug-12	85	4281	1	1	1	1	1
Chart_123	Hospital_7	M	Sep-12	Sep-12	95	4280	1	1	1	2	1
Chart_124	Hospital_7	M	Oct-12	Oct-12	98	4280	1	1	1	2	1
Chart_125	Hospital_7	M	Jun-13	Jun-13	81	4280	1	1	2	0	1
Chart_126	Hospital_7	M	Aug-13	Aug-13	87	4280	1	1	2	0	1
Chart_127	Hospital_7	F	Oct-13	Oct-13	77	4280	1	1	1	2	1
Chart_128	Hospital_7	M	Jan-14	Jan-14	87	4280	1	1	1	2	1
Chart_129	Hospital_7	M	Apr-14	Apr-14	84	4280	1	1	1	1	1
Chart_130	Hospital_7	M	Aug-14	Aug-14	88	4280	1	1	1	0	1

Legend: 0=no; 1=yes; 2=not reported; n.a.=clinical chart not available

Non-cases

Number Clinical Chart	Hospital	Gender	Admission date	Discharge date	Patient's age on admission	Primary Diagnosis (ICD-9)	Non-cases group for myocardial infarction - Validation	Non-cases group for AF/flutter - Validation	Non-cases group for heart failure - Validation
Chart_1	Hospital_1	M	Feb-12	Feb-12	--	4275	n.a.	n.a.	n.a.
Chart_2	Hospital_1	F	Feb-12	Mar-12	89	431	1	1	1
Chart_3	Hospital_1	M	Jul-12	Jul-12	75	41401	1	1	1
Chart_4	Hospital_1	M	Aug-12	Aug-12	58	43401	1	1	1
Chart_5	Hospital_1	F	Sep-12	Sep-12	55	41401	1	1	1
Chart_6	Hospital_1	M	Sep-12	Oct-12	88	431	1	1	1
Chart_7	Hospital_1	F	Nov-12	Nov-12	82	4412	1	1	0
Chart_8	Hospital_1	M	Nov-12	Nov-12	60	41401	1	1	1
Chart_9	Hospital_1	F	Dec-12	Dec-12	65	42651	1	0	1
Chart_10	Hospital_1	M	Feb-13	Feb-13	77	431	1	1	1
Chart_11	Hospital_1	M	Feb-13	Feb-13	85	41400	1	1	1
Chart_12	Hospital_1	M	Apr-13	Apr-13	72	4241	1	1	1
Chart_13	Hospital_1	M	Apr-13	May-13	82	4512	1	0	0
Chart_14	Hospital_1	F	Jun-13	Jun-13	60	4139	1	1	1
Chart_15	Hospital_1	F	Jun-13	Jun-13	78	4111	1	1	1
Chart_16	Hospital_1	F	Jun-13	Jun-13	87	4552	1	1	1
Chart_17	Hospital_1	F	Jul-13	Jul-13	--	45119	n.a.	n.a.	n.a.
Chart_18	Hospital_1	M	Jul-13	Jul-13	83	42781	1	1	1
Chart_19	Hospital_1	F	Sep-13	Sep-13	79	4580	1	1	1
Chart_20	Hospital_1	M	Sep-13	Sep-13	78	431	1	1	1
Chart_21	Hospital_1	M	Sep-13	Oct-13	83	43491	1	1	1
Chart_22	Hospital_1	M	Jan-14	Jan-14	80	4321	1	1	1
Chart_23	Hospital_1	M	Feb-14	Feb-14	75	42789	1	1	1
Chart_24	Hospital_1	M	Feb-14	Mar-14	75	4139	1	1	1
Chart_25	Hospital_1	M	Mar-14	Mar-14	94	45829	1	1	1
Chart_26	Hospital_1	M	Mar-14	Apr-14	--	4239	n.a.	n.a.	n.a.
Chart_27	Hospital_1	M	Mar-14	Apr-14	86	43491	1	1	1
Chart_28	Hospital_1	F	Jun-14	Jun-14	20	4372	1	1	1
Chart_29	Hospital_1	M	Jul-14	Jul-14	79	42781	1	1	1
Chart_30	Hospital_1	M	Jul-14	Jul-14	84	44422	1	1	1
Chart_31	Hospital_1	M	Sep-14	Sep-14	74	41401	1	1	0

Number Clinical Chart	Hospital	Gender	Admission date	Discharge date	Patient's age on admission	Primary Diagnosis (ICD-9)	Non-cases group for myocardial infarction - Validation	Non-cases group for AF/flutter - Validation	Non-cases group for heart failure - Validation
Chart_32	Hospital_1	F	Oct-14	Oct-14	81	4260	1	1	1
Chart_33	Hospital_1	M	Nov-14	Nov-14	76	41189	0	1	1
Chart_34	Hospital_2	F	Jan-12	Jan-12	83	40291	1	1	1
Chart_35	Hospital_2	F	Mar-12	Mar-12	78	43400	1	1	1
Chart_36	Hospital_2	M	Apr-12	Apr-12	69	41401	1	1	1
Chart_37	Hospital_2	M	Apr-12	Apr-12	32	4555	1	1	1
Chart_38	Hospital_2	F	Jul-12	Jul-12	77	4254	1	1	1
Chart_39	Hospital_2	F	Sep-12	Sep-12	78	43401	1	1	1
Chart_40	Hospital_2	F	Dec-12	Dec-12	64	41401	1	1	1
Chart_41	Hospital_2	F	Dec-12	Dec-12	79	431	1	1	1
Chart_42	Hospital_2	M	Feb-13	Feb-13	84	40210	1	1	1
Chart_43	Hospital_2	M	Feb-13	Feb-13	66	4149	1	1	1
Chart_44	Hospital_2	M	Apr-13	May-13	73	45119	1	1	1
Chart_45	Hospital_2	F	Dec-13	Dec-13	87	3968	1	1	1
Chart_46	Hospital_2	F	Jan-14	Jan-14	63	4541	1	1	1
Chart_47	Hospital_2	F	Apr-14	Apr-14	57	41401	1	1	1
Chart_48	Hospital_2	M	Apr-14	May-14	83	4240	1	1	1
Chart_49	Hospital_2	M	May-14	May-14	64	4149	1	1	1
Chart_50	Hospital_2	F	Aug-14	Aug-14	88	43401	1	1	1
Chart_51	Hospital_2	F	Aug-14	Aug-14	75	43401	1	1	1
Chart_52	Hospital_2	F	Oct-14	Oct-14	72	43400	1	1	1
Chart_53	Hospital_2	M	Oct-14	Oct-14	81	4358	1	1	1
Chart_54	Hospital_2	F	Oct-14	Oct-14	75	4321	1	1	1
Chart_55	Hospital_3	F	Aug-12	Aug-12	--	42760	n.a.	n.a.	n.a.
Chart_56	Hospital_3	M	Dec-12	Dec-12	47	4552	1	1	1
Chart_57	Hospital_3	M	May-13	Jun-13	85	43401	1	1	1
Chart_58	Hospital_3	F	Nov-13	Nov-13	81	45119	1	1	1
Chart_59	Hospital_3	M	May-14	May-14	91	43401	1	1	1
Chart_60	Hospital_4	F	Sep-12	Oct-12	92	44024	1	1	1
Chart_61	Hospital_4	F	Dec-12	Dec-12	62	43411	1	0	1
Chart_62	Hospital_4	M	Dec-12	Dec-12	79	43401	1	1	1
Chart_63	Hospital_4	M	Jul-13	Aug-13	87	42781	1	1	1
Chart_64	Hospital_5	F	May-12	May-12	63	4465	1	1	1

Number Clinical Chart	Hospital	Gender	Admission date	Discharge date	Patient's age on admission	Primary Diagnosis (ICD-9)	Non-cases group for myocardial infarction - Validation	Non-cases group for AF/flutter - Validation	Non-cases group for heart failure - Validation
Chart_65	Hospital_5	F	Jul-12	Jul-12	81	43400	1	1	1
Chart_66	Hospital_5	M	Oct-12	Oct-12	76	4241	1	1	0
Chart_67	Hospital_5	M	Nov-12	Nov-12	55	4552	1	1	1
Chart_68	Hospital_5	M	Nov-12	Dec-12	68	4148	1	1	1
Chart_69	Hospital_5	M	Jun-13	Jun-13	68	4241	1	1	1
Chart_70	Hospital_5	F	Jul-13	Jul-13	80	4139	0	1	1
Chart_71	Hospital_5	F	May-14	May-14	78	4260	1	1	1
Chart_72	Hospital_5	M	Jul-14	Jul-14	87	4358	1	0	1
Chart_73	Hospital_6	M	Mar-12	Apr-12	76	4111	1	1	1
Chart_74	Hospital_6	M	Jul-12	Jul-12	84	43401	1	1	1
Chart_75	Hospital_6	F	Oct-12	Oct-12	75	43401	1	1	1
Chart_76	Hospital_6	F	Nov-13	Dec-13	87	43411	1	0	1
Chart_77	Hospital_6	M	Mar-14	Mar-14	68	43400	1	1	1
Chart_78	Hospital_7	F	Jul-12	Jul-12	90	4321	1	0	0
Chart_79	Hospital_7	F	Feb-13	Feb-13	47	4549	1	1	1
Chart_80	Hospital_7	M	Jul-14	Jul-14	39	42099	1	1	1

Legend: 0=no; 1=yes; 2=not reported; n.a.=clinical chart not available
